# Clinical and epidemiological characteristics of patients seeking COVID-19 testing in a private centre in Malaysia: Is there a role for private healthcare in battling the outbreak?

**DOI:** 10.1371/journal.pone.0258671

**Published:** 2021-10-14

**Authors:** Yock Ping Chow, Brenda Huey Zien Chin, Jin Ming Loo, Loshini R. Moorthy, Jamuna Jairaman, Lian Huat Tan, Wendy Wan Ying Tay

**Affiliations:** 1 Clinical Research Centre, Sunway Medical Centre, Selangor Darul Ehsan, Malaysia; 2 Department of Information & Communication Technologies, Sunway Medical Centre, Selangor Darul Ehsan, Malaysia; 3 Department of Diagnostic Laboratory, Sunway Medical Centre, Selangor Darul Ehsan, Malaysia; 4 Department of Medicine, Sunway Medical Centre, Selangor Darul Ehsan, Malaysia; Istanbul University Istanbul Faculty of Medicine: Istanbul Universitesi Istanbul Tip Fakultesi, TURKEY

## Abstract

**Objective:**

This cross-sectional observational study summarized the baseline characteristics of subjects who underwent COVID-19 molecular testing in a private medical centre located in the state of Selangor in Malaysia between 1 Oct 2020 and 31 Jan 2021. We compared the baseline characteristics between subjects who were tested positive and negative of SARS-CoV-2 infection, and identified risk factors which may be predictive of SARS-CoV-2 positivity.

**Methods and findings:**

A total of 36603 subjects who were tested for COVID-19 infection via molecular assays at Sunway Medical Centre between Oct 1, 2020 and Jan 31, 2021, and consented to participate in this observation study were included for analysis. Descriptive statistics was used to summarize the study cohort, whereas logistic regression analysis was used to identify risk factors associated with SARS-CoV-2 positivity. Among the reasons listed for COVID-19 screening were those who needed clearance for travelling, clearance to return to work, or clearance prior to hospital admission. They accounted for 67.7% of tested subjects, followed by the self-referred group (27.3%). Most of the confirmed cases were asymptomatic (62.6%), had no travel history (99.6%), and had neither exposure to SARS-CoV-2 confirmed cases (61.9%) nor exposure to patients under investigation (82.7%) and disease clusters (89.2%). Those who presented with loss of smell or taste (OR: 26.91; 95% CI: 14.81–48.92, p<0.001), fever (OR:3.97; 95% CI: 2.54–6.20, p<0.001), running nose (OR: 1.75; 95% CI:1.10–2.79, p = 0.019) or other symptoms (OR: 5.63; 95% CI:1.68–18.91, p = 0.005) were significantly associated with SARS-CoV-2 positivity in the multivariate logistic regression analysis.

**Conclusion:**

Our study showed that majority of patients seeking COVID-19 testing in a private healthcare setting were mainly asymptomatic with low epidemiological risk. Consequently, the average positivity rate was 1.2% compared to the national cumulative positivity rate of 4.65%. Consistent with other studies, we found that loss of smell or taste, fever and running nose were associated with SARS-CoV-2 positivity. We believe that strengthening the capacity of private health institutions is important in the national battle against the COVID-19 pandemic, emphasizing the importance of public-private partnership to improve the quality of clinical care.

## Introduction

As of 28 March 2021, COVID-19 had spread to 223 countries; infecting more than 126 million people and causing more than 2.7 million deaths worldwide. Of the regions in the world, America had the highest number of confirmed COVID-19 cases (n = 55243776) followed by Europe (n = 44181716), South-East Asia (n = 4619886), the Eastern Mediterranean (7392128), Africa (3061438), and the Western Pacific (n = 1859851) (https://covid19.who.int/). The number of confirmed COVID-19 cases in Malaysia had risen to 341944 cases with 1255 deaths. Among the 14 states in Malaysia, Selangor was the most affected, with 122934 confirmed cases as of 23th March 2021 [1].

According to the guidelines set by the Ministry of Health Malaysia (MOH), the diagnosis of COVID-19 is made by the detection of SARS-CoV-2 via molecular-based testing. In the beginning of the COVID-19 pandemic, there were only 23 laboratories nationwide which offered up to a total of 1000 RT-PCR tests daily. Within a year’s time, Malaysia had increased the testing capacity to ~70000 tests per day, with the support of 67 laboratories nationwide. Sunway Medical Centre’s Molecular Diagnostic Laboratory is one of the pioneer private-hospital-based laboratories certified by the MOH to offer COVID-19 screening services. To supplement testing efforts, a digital online screening form was established to ascertain demographics, presence of clinical symptoms, and other clinically relevant information from tested individuals. The digital platform served as a systematic primary source for supporting research needs, and enabled more efficient contract tracing.

This cross-sectional observational study summarized the baseline characteristics of subjects who underwent COVID-19 molecular testing in a private medical centre located in the state of Selangor in Malaysia between 1 Oct 2020 and 31 Jan 2021, compared baseline characteristics between subjects who were tested positive and negative of SARS-CoV-2 infection, and identified risk factors which may be predictive of SARS-CoV-2 positivity.

## Methodology

### Study design and data collection

This cross-sectional study formed part of an ongoing prospective observational study to collect data from individuals who tested for SARS-CoV-2 infection at Sunway Medical Centre, starting from Oct 1, 2020. The study protocol was approved by the Sunway Independent Research Ethics Committee [SREC Reference Number: 017/2020/IND/ER]. To manage the high demand of COVID-19 testing, a digital COVID-19 screening form was made available to collect the demographic data, epidemiological exposure history (travel, close contact with confirmed SARS-CoV-2, patient under investigation or disease cluster/hotspot), and clinical characteristics of tested individuals. The requirement for written informed consent was waived by the independent ethics committee. However, individuals who agreed to contribute their data for research purposes were asked to provide electronic consent by ticking the “consent for research” box on the digital screening form.

To preserve data quality, the digital COVID-19 screening form was developed, validated and pretested by a group of 28 people [healthcare professionals (consultant of infectious diseases, attending medical doctors in the emergency department), laboratory personnel, legal, management, marketing and information technology team] before field administration. To minimize reporting bias, the questions were presented in both English and Chinese in the survey link. The first section consisting of basic sociodemographic information were filled up by subjects themselves, whereas the second section consisting of clinical information were filled up by healthcare professionals (attending medical doctors). To minimize selection bias, all subjects who tested within the study period and consented were included into this study.

This report described the demographics, history of exposure, reason for testing, and clinical characteristics of subjects who came for molecular testing of SARS-CoV-2 infection at Sunway Medical Centre between Oct 1, 2020 and Jan 31, 2020. The inclusion criteria were: (1) Diagnosis of COVID-19 was confirmed through real-time reverse-transcriptase-polymerase-chain-reaction (RT–PCR) assays of combination of nasopharyngeal and oropharyngeal swabs, in accordance to guidance from the Ministry of Health Malaysia; (2) Subjects agreed to participate in this study. The exclusion criteria were: (1) Subject with incomplete data, (2) Subject who did not agree to participate in this study. All data were extracted from the laboratory information system and the COVID-19 electronic screening database. The data were anonymized prior to analysis.

### SARS-CoV-2 infection testing

A total of 2 swabs (nasopharyngeal and oropharyngeal) were taken from each patient by medical doctors who had undergone training and certification by the Ministry of Health Malaysia to perform COVID-19 swabbing. The swab samples were then placed into a single tube of viral transport medium immediately after sampling and were kept in storage box containing ice packs. The samples were then sent to Molecular Diagnostics Laboratory of Sunway Medical Centre which has been certified by Ministry of Health Malaysia to perform the COVID molecular testing [2]. The test results were released to subjects within 24 hours of testing.

All the samples in this study were tested using Allplex 2019-nCoV Assay (Seegene Inc., Seoul, South Korea) for the detection of genes encoding the SARS-CoV-2 envelope protein (E), RNA-dependent RNA polymerase (RdRp), and nucleocapsid protein (N). The sample was considered SARS-CoV-2 positive if two of the three genes showed amplification with Ct ≤ 40, whereas sample was considered SARS-CoV-2 negative if the internal control was amplified but not the three target genes or if there is only single gene detected.

### Statistical analysis

The Kolmogorov–Smirnov test was used to test the distribution of the continuous variables (age and interval between symptom onset and testing). Both age and interval (days) from symptom onset to diagnosis were not normally distributed, and were described as median with range values. Mann-Whitney test was used to compare the continuous variables. Chi-square or Fisher’s exact test was used to compare the categorical variables. Univariate and multivariate logistic regression analyses were conducted to identify the risk factors for SARS-CoV-2 test positivity. Variables from the univariate analysis with a *p*-value <0.05 were included in the multivariate logistic regression analysis. Variables retaining *p*-value <0.05 in multivariate analysis were considered statistically significant. All statistical analyses were performed using Statistical Package for the Social Sciences (SPSS) version 25.0 software.

## Result

### Baseline characteristics of patients tested for SARS-CoV-2 infection

**Fig 1:**
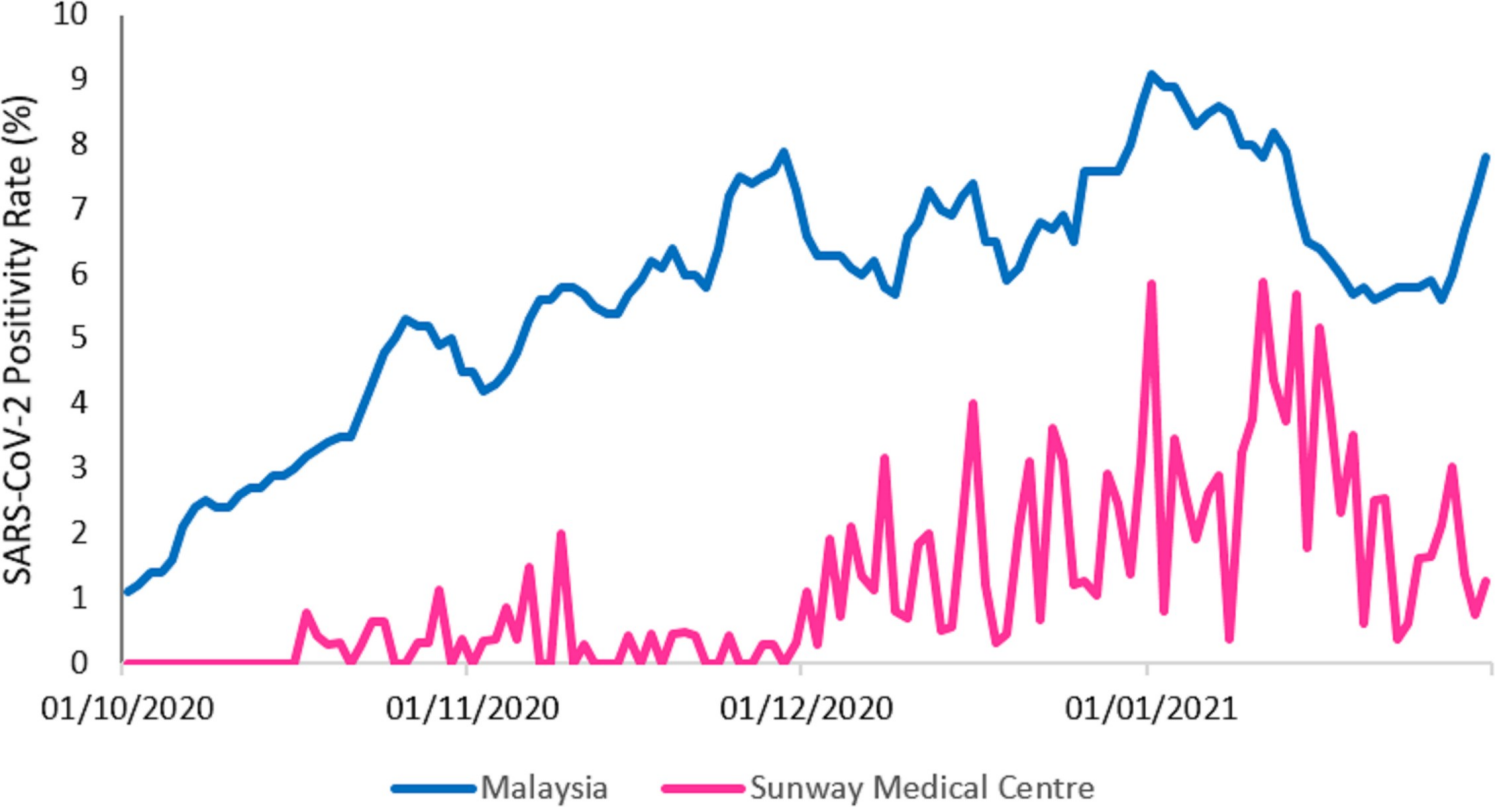
SAR-CoV-2 positivity rate of our study cohort (Sunway Medical Centre) and national data between Oct 1, 2020 and Jan 31, 2021.

A total of 36,603 subjects who tested for SARS-CoV-2 infection between Oct 1, 2020 and January 31, 2021 consented to participate in this study. The SARS-CoV-2 positivity rate of our cohort as compared to national data over the 4-month study period was shown in Fig 1. Overall, the positivity rate of our study cohort was much lower compared to national statistics (average 1.21% vs 5.78%). In our centre, the average positivity rate was shown to increase from 0.18% and 0.31% in October and November 2020 to 1.72% and 2.68% in December and January respectively. Similar pattern was observed in our national data, which recorded the spike in December (6.77%) and January (7.13%) as compared to October (3.24%) and November (5.98%).

The details of the study cohort were summarized in Table 1. The tested individuals had a median age of 33 years [range 0 to 96 years]. Most of the tested subjects were Malaysian (n = 27044, 73.9%), and male (n = 22411, 61.2%). Chinese (n = 19680, 53.8%) represented the most tested ethnicity group, followed by Malay (n = 7194, 19.7%), other ethnicities (n = 6628, 18.1%), and Indian (n = 3101, 8.5%). Majority had no travel history (n = 36439, 99.6%), and had no close contact with confirmed SAR-CoV-2 cases (n = 32143, 87.8%), patients under investigation (n = 34395, 94.0%) or disease clusters (n = 34997, 95.6%). Nearly 1/3 subjects sought testing to fulfill pre-travel requirements (n = 11292, 30.8%). Self-referred subjects represented the second largest group for testing (n = 10003, 27.3%), followed by those required by their employer (n = 7159, 19.6%), pre-admission patients (n = 6331, 17.3%), on-site/workplace workers (n = 1211, 3.3%), those mandated by health authorities (n = 538, 1.5%), and those being attended to at Accident & Emergency (n = 69, 0.2%). Nearly 97% of the tests were for diagnostic purposes.

**Table 1:** Epidemiological and clinical characteristics of subjects who tested positive for SARS-CoV-2 infection vs subjects who tested negative for SARS-CoV-2 infection at Sunway Medical Centre between Oct 1, 2020 and Jan 31, 2021.

Variable	All cases	Positive	Negative	p-value
	(n,%)	(n,%)	(n,%)	
**Total number of cases**	36603	473 (1.3)	36130 (98.7)	
**Age (years, median (range))**	33 (0–96)	32 (1–84)	33 (0–96)	0.388
**Time interval between symptom onset and testing, median (range)**	2 (0–61)	2 (0–22)	2 (0–61)	0.618
**Age Group**				
≤9	434 (1.2)	13 (2.8)	421 (1.2)	0.155
10–19	1393 (3.8)	19 (4.0)	1374 (3.8)	
20–29	12090 (33.1)	153 (32.4)	11937 (33.1)	
30–39	11009 (30.2)	145 (30.7)	10864 (30.1)	
40–49	6064 (16.6)	69 (14.6)	5995 (16.6)	
50–59	3380 (9.3)	47 (10.0)	3333 (9.2)	
60–69	1591 (4.4)	19 (4.0)	1572 (4.4)	
70–79	519 (1.4)	7 (1.5)	512 (1.4)	
≥80	27 (0.1)	0 (0)	27 (0.1)	
**Gender**				0.026
Male	22411 (61.2)	266 (56.2)	22145 (61.3)	
Female	14192 (38.8)	207 (43.8)	13985 (38.7)	
**Nationality**				<0.0001
Malaysian	27044 (73.9)	434 (91.8)	26610 (73.7)	
Other	9559 (26.1)	39 (8.2)	9520 (26.3)	
**Race**				<0.0001
Chinese	19680 (53.8)	225 (47.6)	19455 (53.8)	
Malay	7194 (19.7)	135 (28.5)	7059 (19.5)	
Indian	3101 (8.5)	70 (14.8)	3031 (8.4)	
Other	6628 (18.1)	43 (9.1)	6585 (18.2)	
**Travel History**				1.00*
Yes	164 (0.4)	2 (0.4)	162 (0.4)	
No	36439 (99.6)	471 (99.6)	35968 (99.6)	
**Close Contact with Confirmed SARS-CoV-2 Cases**				<0.0001
Yes	4460 (12.2)	180 (38.1)	4280 (11.8)	
No	32143 (87.8)	293 (61.9)	31850 (88.2)	
**Close Contact with Patient Under Investigation**				<0.001
Yes	2208 (6.0)	82 (17.3)	2126 (5.9)	
No	34395 (94.0)	391 (82.7)	34004 (94.1)	
**Close Contact with Disease Cluster/Hotspot**				<0.001
Yes	1606 (4.4)	51 (10.8)	1555 (4.3)	
No	34997 (95.6)	422 (89.2)	34575 (95.7)	
***Reason for Screening***				<0.001
Accident & Emergency	69 (0.2)	1 (0.2)	68 (0.2)	
Admission to hospital	6331(17.3)	17 (3.6)	6314 (17.5)	
Employer requirement	7159 (19.6)	69 (14.6)	7090 (19.6)	
On-Site/Workplace screening	1211 (3.3)	7 (1.5)	1204 (3.3)	
Pre Travel Requirement	11292 (30.8)	20 (4.2)	11272 (31.2)	
Requirement from health	538 (1.5)	12 (2.5)	526 (1.5)	
authority				
Self-referral–Walk in/Drive	10003 (27.3)	347 (73.4)	9656 (26.7)	
Thru/Home Screening				
***Purpose of Sampling***				0.662*
Advanced testing	12 (0.03)	0 (0)	12 (0.03)	
Cluster	9 (0.02)	0 (0)	9 (0.02)	
COVID-19 Surveillance	1222 (3.34)	18 (3.81)	1204 (3.33)	
Diagnostic	35354 (96.6)	455 (96.2)	34899 (96.6)	
Referral Programme	3 (0.01)	0 (0)	3 (0.01)	
Research	3 (0.01)	0 (0)	3 (0.01)	
***Symptoms category***				<0.001
Asymptomatic	34679 (94.7)	296 (62.6))	34383 (95.2)	
Symptomatic	1924 (5.3)	177 (37.4)	1747 (4.8)	
***Presenting Symptoms***				
Cough	652 (1.8)	63 (13.3)	589 (1.6)	<0.001
Cough (No)	35951 (98.2)	410 (86.7)	35541 (98.4)	
Chest Pain	10 (0.03)	0 (0)	10 (0.03)	1.00*
Chest Pain (No)	36593 (99.97)	473 (100)	36120 (99.97)	
Running Nose	491 (1.3)	51 (10.8)	440 (1.2)	<0.001
Running Nose (No)	36112 (98.7)	422 (89.2)	35690 (98.8)	
Fever	439 (1.2)	66 (14.0)	373 (1.0)	<0.001
Fever (No)	36164 (98.8)	407 (86.0)	35757 (99.0)	
Sore Throat	749 (2.0)	63 (13.3)	686 (1.9)	<0.001
Sore Throat (No)	35854 (98)	410 (86.7)	35444 (98.1)	
Myalgia	42 (0.1)	9 (1.9)	33 (0.1)	<0.001*
Myalgia (No)	36561 (99.9)	464 (98.1)	36097 (99.9)	
Gastrointestinal Symptom	40 (0.1)	0 (0)	40 (0.1)	1.00*
Gastrointestinal Symptom (No)	36563 (99.9)	473 (100)	36090 (99.9)	
Loss of Smell and Taste	74 (0.2)	44 (9.3)	30 (0.1)	<0.001*
Loss of Smell and Taste (No)	36529 (99.8)	429 (90.7)	36100 (99.9)	
Nausea or Vomiting	47 (0.1)	3 (0.6)	44 (0.1)	0.023*
Nausea or Vomiting (No)	36556 (99.9)	470 (99.4)	36086 (99.9)	
Fatigue	33 (0.1)	8 (1.7)	25 (0.1)	<0.001*
Fatigue (No)	36570 (99.9)	465 (98.3)	36105 (99.9)	
Shortness of Breath	48 (0.1)	1 (0.2)	47 (0.1)	0.465*
Shortness of Breath (No)	36555 (99.9)	472 (99.8)	36083 (99.9)	
Other Symptoms	25 (0.1)	5 (1.0)	20 (0.1)	<0.001*
Other Symptoms (No)	36578 (99.9)	468 (99.0)	36110 (99.9)	

P < 0.05 was considered statistically significant.

* indicated P-values was obtained by Fisher exact test.

A total of 473 (1.3%) patients were tested positive for SARS-CoV-2 infection, with a median age of 32 years (range 1 to 84 years). Of all SAR-CoV-2-confirmed patients, most were Malaysian (91.8%, *p*<0.0001), and male (56.2%, *p* = 0.026). Nearly half of the individuals with laboratory-confirmed SARS-CoV-2 infection were Chinese (47.6%), followed by Malay (28.5%), Indian (14.8%) and other ethnicities (9.1%). The largest proportion of the infected patients were aged between 20–29 years (32.4%) and 30–39 years (30.7%). Only 6.8% of the patients were below 20 years of age. Regarding the history of exposure, 180 patients (38.1%, *p*<0.0001) had come in contact with SAR-CoV-2-confirmed cases, 82 patients (17.3%, p<0.001) had come in contact with patients under investigation, 51 patients (10.8%, p<0.001) had come in contact with a disease cluster/hotspot. Only 2 patients (0.4%, p = 1.0) reported they had local travel history. Asymptomatic cases accounted for 63% (p<0.001) of the positive cases. For symptomatic patients, fever (n = 66, 14.0%, p<0.001), cough (n = 63, 13.3%, p<0.001), sore throat (n = 63, 13.3% p<0.001), running nose (n = 51, 10.8%, p<0.001), and loss of smell and taste (n = 44, 9.3%, p<0.001) were the five most common reported symptoms. Less common symptoms were myalgia (n = 9, 1.9%, p<0.001), nausea or vomiting (n = 3, 0.6%, p = 0.023), fatigue (n = 8, 1.7%, p<0.001), shortness of breath (n = 1, 0.2%, p = 0.465), and other symptoms (n = 5, 1.0%, p<0.001). None of the confirmed SARS-CoV-2 infected patients presented with gastrointestinal symptoms or chest pain. The median duration from onset of symptoms to diagnosis was 2 days (range 0–22 days).

The positivity rate by reason of screening was summarized in S1 Table. Self-referral group had the highest positivity rate (n = 347, 3.47%), followed by those mandated by health authority (n = 12, 2.23%). One patient who presented to accident and emergency department tested positive during the study period. The remaining testing groups had positivity rate below 1%. Both pre-travel, and pre-admission testing groups had the lowest positivity rate, with 0.18% (n = 20) and 0.27% (n = 17) respectively.

### Predictors for SARS-CoV-2 test positivity

Univariate and multivariate logistic regression models were performed to identify potential risk factors for SARS-CoV-2 test positivity. In the univariate analysis (Table 2), the odds of positive SARS-CoV-2 testing was 19% (OR:0.81; 95% CI: 0.68–0.97) lower in males. Malaysians had higher odds of testing positive than other nationalities (OR: 3.98; 95% CI: 2.87–5.53). In comparison with other ethnicities, the Chinese (OR: 1.77; 95% CI: 1.28–2.46), Malays (OR: 2.93; 95% CI: 2.07–4.14) and Indians (OR: 3.54; 95% CI: 2.41–5.18) showed increased odds of testing positive for SARS-CoV-2 infection. Those who had close contact with confirmed SARS-CoV-2 cases (OR:4.57; 95% CI:3.79–5.52), patients under investigation (OR:3.35; 95% CI:2.63–4.27) or a disease cluster/hotspot (OR:2.69; 95% CI: 3.00–3.61) were found significantly associated with SARS-CoV-2 infectivity. The odds of positive SARS-CoV-2 testing was 11.77 times higher in symptomatic patients (OR: 11.77, 95% CI:9.71–14.27). Pertaining to clinical signs and symptoms, individuals who experienced loss of smell and taste (OR: 123.42; 95% CI: 76.85–198.21) had the highest odds of COVID-19 positivity, followed by fatigue (OR: 24.85; 95% CI:11.15–55.37), myalgia (OR: 21.22; 95% CI: 10.10–44.59), other symptoms (OR: 19.29; 95% CI: 7.21–51.61), fever (OR: 15.55; 95% CI: 11.76–20.56), running nose (OR: 9.80; 95% CI: 7.22–13.30), cough (OR: 9.27; 95% CI: 7.03–12.24), sore throat (OR: 7.94; 95% CI: 6.03–10.46), and nausea or vomiting (OR: 5.24; 95% CI: 1.62–16.92). Those with a longer time interval between symptom onset and testing had ~5% increased risk of testing positive (OR: 1.05; 95% CI:1.00–1.09), but with marginally significant p-value (p = 0.046).

**Table 2:** Univariate logistic regression analysis of risk factors associated with SARS-CoV-2 test positivity.

Variable	Univariate logistic regression	
	OR (95% CI)	p-value
**Age**	1.00 (0.99–1.00)	0.245
**Time interval between symptom onset and testing**	1.05 (1.00–1.09)	0.046
***Gender (Male)***	0.81 (0.68–0.97)	0.025
**Nationality (Malaysian)**	3.98 (2.87–5.53)	<0.001
**Race**		
Chinese	1.77 (1.28–2.46)	0.001
Malay	2.93 (2.07–4.14)	<0.001
Indian	3.54 (2.41–5.18)	<0.001
Other	1 [Reference]	
**Travel History**	0.94 (0.23–3.81)	0.934
**Close Contact with Confirmed SARS-CoV-2 Cases**	4.57 (3.79–5.52)	<0.001
**Close Contact with Patient Under Investigation**	3.35 (2.63–4.27)	<0.001
**Close Contact with Disease Cluster/Hotspot**	2.69 (3.00–3.61)	<0.001
***Reason for Screening***		
Accident & Emergency	1 [Reference]	
Admission to hospital	0.183 (0.024–1.395)	0.101
Employer requirement	0.662 (0.091–4.834)	0.684
On-Site/Workplace screening	0.395 (0.048–3.259)	0.389
Pre Travel Requirement	0.121 (0.016–0.912)	0.04
Requirement from health authority	1.551 (0.199–12.118)	0.675
Self-referral–Walk in/Drive Thru/Home Screening	2.444 (0.338–17.65)	0.376
***Purpose of Sampling***		
Advanced testing	0.998	1
Cluster	0.998	1
COVID-19 Surveillance	24103874.63	1
Diagnostic	21020316.72	0.999
Referral Programme	0.998	0.999
Research	1 [Reference]	1
**Symptomatic**	11.77 (9.71–14.27)	<0.001
***Presenting Symptoms***		
Cough	9.27 (7.03–12.24)	<0.001
Chest Pain	0	0.999
Running Nose	9.80 (7.22–13.30)	<0.001
Fever	15.55 (11.76–20.56)	<0.001
Sore Throat	7.94 (6.03–10.46)	<0.001
Myalgia	21.22 (10.10–44.59)	<0.001
Gastrointestinal Symptom	0	0.998
Loss of Smell and Taste	123.42 (76.85–198.21)	<0.001
Nausea or Vomiting	5.24 (1.62–16.92)	0.006
Fatigue	24.85 (11.15–55.37)	<0.001
Shortness of Breath	1.63 (0.22–11.81)	0.63
Other Symptoms	19.29 (7.21–51.61]	<0.001

OR: adjusted odd ratio.

*P < 0.05 was considered statistically significant.

Subsequently, variables with statistical significance in the univariate analysis were subjected to multivariate logistic regression analysis (Table 3). Male (OR:1.79; 95% CI: 1.24–2.58), close contact with confirmed COVID-19 patient (OR:3.28; 95% CI: 2.12–5.07), and presentation at diagnosis with fever (OR:3.97; 95% CI: 2.54–6.20), running nose (OR: 1.75; 95% CI:1.10–2.79), loss of smell and taste (OR: 26.91; 95% CI: 14.81–48.92), and other symptoms (OR: 5.63; 95% CI:1.68–18.91), time interval between symptom onset and testing remained significantly associated with increased odds of testing positive (OR: 1.06; 95% CI: 1.00–1.11). Loss of smell or taste had the strongest association with SARS-CoV-2 positivity. In contrast, the Chinese (OR:0.43; 95% CI: 0.19–0.99) and Malays (OR:0.33; 95% CI: 0.14–0.77) appeared to have a lower risk of SARS-CoV-2 positivity as compared to other ethnicities.

**Table 3:** Multivariate logistic regression analysis of risk factors associated with SARS-CoV-2 test positivity.

Variable	Multivariate logistic regression	
	OR (95% CI)	p-value
**Time interval between symptom onset and testing (Day)**	1.06 (1.00–1.11)	0.036*
**Gender (Male)**	1.79 (1.24–2.58)	0.002*
**Nationality (Malaysian)**	3.05 (0.89–10.44)	0.076
**Race**		
Chinese	0.43 (0.19–0.99)	0.048*
Malay	0.33 (0.14–0.77)	0.011*
Indian	0.55 (0.22–1.35)	0.191
Other		
**Close Contact with Confirmed SARS-CoV-2 Cases**	3.28 (2.12–5.07)	<0.001*
**Close Contact with Patient Under Investigation**	1.53 (0.89–2.63)	0.122
**Close Contact with Disease Cluster/Hotspot**	1.08 (0.59–1.97)	0.8
***Presenting Symptoms***		
Cough	1.38 (0.94–2.03)	0.104
Running Nose	1.75 (1.10–2.79)	0.019*
Fever	3.97 (2.54–6.20)	<0.001*
Sore Throat	1.16 (0.79–1.69)	0.458
Myalgia	1.81 (0.71–4.64)	0.218
Loss of Smell and Taste	26.91 (14.81–48.92)	<0.001*
Nausea or Vomiting	0.83 (0.19–3.61)	0.798
Fatigue	1.22 (0.35–4.25)	0.758
Other Symptoms	5.63 (1.68–18.91)	0.005*

OR: *odd ratio*.

*P < 0.05 was considered statistically significant.

## Discussion

In Malaysia, a nationwide observational study was conducted to describe the clinical characteristics of hospitalized COVID-19 patients and the risk factors associated with disease severity [3]. However, there is sparse information regarding the demographics, history of epidemiological exposure and clinical presentation of subjects who sought COVID-19 testing in Malaysia. To the best of our knowledge, this study is the first to describe the demographics and baseline characteristics of the outpatient cohort testing for SARS-CoV-2 infection in a private-hospital-based laboratory in Malaysia. Our study demonstrated that majority of individuals who came for testing at our centre were of lower risk groups who had no history of epidemiological exposure and no COVID-19-related symptoms; in our cohort, 89% reported no close contact with confirmed SARS-CoV-2 cases, and around 95% reported no close contact with patients under investigation and/or disease cluster/hotspot areas. On top of that, 95% reported no symptoms. We believe our study demonstrated the role of private hospitals in complementing the efforts of the public sector, especially when it comes to catering to healthcare demands in a holistic manner. Up till the conclusion of this research project, Ministry of Health Malaysia implemented targeted screening on symptomatic subjects and/or direct contacts of confirmed SARS CoV-2 patients and those who were exposed to a known COVID-19 cluster or outbreak [4–6]. Therefore, individuals not fulfilling criteria above, who required or desired testing due to other reasons were mainly provided for by private healthcare providers.

Over the period of four months, from October 1, 2020 to January 31, 2021, more than 36,000 individuals sought testing in Sunway Medical Centre. Our study showed that among them, 30% did so due to pre-travel requirements, as imposed by various governments and the airline industry for international air travel. The demand for prompt results was particularly high in this group as most airlines considered results valid for only up till 72 hours before boarding. In addition, 27% of the tested subjects were self-referred. These could be among the ones who had been indirectly exposed to COVID-19 patients yet did not fulfill the Ministry of Health’s criteria or definition of direct contact [7]. Some of them may had also been alerted by “My Sejahtera”, a digital application launched by the government of Malaysia to assist in monitoring the COVID-19 outbreak. Through this app, users were alerted if the premises they had visited reported COVID-19 positive cases.

Furthermore, 20% of tested individuals sought testing due to employers’ requirements. As the outbreak progressed, the government of Malaysia made employers accountable for the safety of their workers by mandating daily surveillance of temperature, symptoms, and risk factors, as well as promoting disease awareness in the workplace. To prevent workplace-linked transmission of COVID-19, Ministry of Health Malaysia encouraged companies to screen their workforce for COVID-19 before returning to work since May 2020 [8]. Upon emergence of workplace clusters amongst foreign workers, the government tightened this recommendation by mandating foreign workers in Malaysia spanning all sectors to be screened for COVID-19 from 1^st^ January 2021 onwards [9]. These measures, in combination with social anxiety surrounding COVID-19 infections had led to employers taking a proactive stance to pay, test and screen their workers for COVID-19. In addition, 17% of individuals sought testing in Sunway Medical Centre due to pre-admission requirements for elective procedures in the hospital. To ensure the safety of healthcare workers, and to prevent spread of COVID-19 in the healthcare setting, patients were required to undergo COVID-19 test before admission.

Our study showed that there was a high incidence of asymptomatic COVID-19 cases detected at our centre at 62.6% which was comparable to the nationwide hospitalized cohort (50.2%) [2]. Moreover, a similarly high proportion of asymptomatic COVID-19 infections (≥ 50%) was observed in cohorts of other countries, such as in Bahrain (50%), South Korea (62%), the United Kingdom (86.1%), and South India (91%) [10–13]. The high proportion of asymptomatic carriers remained a major challenge to contain the transmission of COVID-19, and had led to missed diagnoses and silent transmissions within the community [14, 15]. Therefore, simple public health measures should continue to be advocated to the public such as practice of wearing face masks, frequent handwashing, use of potent surface disinfectants, and adequate physical distancing combined with effective ventilation, should remain as the primary preventive measures [16, 17].

The U.S. Centers for Disease Control and Prevention recognized fever or chills, cough, shortness of breath (dyspnea), fatigue, myalgia, headache, sore throat, runny nose, nausea or vomiting, gastrointestinal issues or diarrhea, and loss of taste or smell as the main clinical symptoms of COVID-19 [18]. Consistent with other studies, our analysis identified fever as the most common symptom in COVID-19 patients (14%), and was associated with SARS-CoV-2 positivity (OR:3.97; 95% CI: 2.54–6.20) [19, 20]. The other statistically significant symptoms were running nose (OR:1.75; 95% CI: 1.10–2.79), loss of smell and taste (OR:26.91; 95% CI: 14.81–48.92), and other symptoms (OR:5.63; 95% CI: 1.68–18.91), although many COVID-19 positive cases also presented with complaints of cough (13.3%) and sore throat (13.3%), which a survey in the United Kingdom found to be more common in the B117 variant of COVID-19 [21].

Distinguishing COVID-19 from other respiratory diseases had been challenging as the presenting symptoms overlap, such as fever, cough, running nose, and sore throat. Simultaneous detection and differentiation of SARS-CoV-2 with other viruses with multiplex assays would be helpful to improve clinical diagnostic efficiency [22–25]. For instance, BD MAX rapid multiplex PCR panel developed by Chung et al. allowed rapid detection of SARS-CoV-2, influenza A/B, and RSV in a single assay [22]. Our laboratory had since started exploring multiplex assay as part of SARS-CoV-2 testing options to aid rapid clinical decision-making.

Symptoms that may be less likely to overlap with other respiratory diseases were loss of smell (anosmia) or taste (ageusia). In our study, anosmia and ageusia were experienced by 9.3% of COVID-19 positive patients as opposed to 0.1% COVID-19 negative patients. Consistent with other studies [26–28], loss of smell or taste had been shown to be a significant predictor of SARS-CoV-2 positivity in our cohort, with an odds ratio of 26. A recent meta-analysis showed that prevalence of smell or taste dysfunction was higher in Europeans (54.4%) and North Americans (51.11%) as compared to Asians (31.39%) and Australians (10.71%) [29]. Ageusia would take from weeks to months to completely resolve [30], we believe further study is warranted to understand the impact of loss of sense of smell and taste in patients especially in Malaysia where food is a critical component in cultural and social interactions.

Malaysia has a dual-tiered system of healthcare services. A government-run and funded public sector under the Ministry of Health, and a fast-growing private sector catering for private insurance patients and patients who pay out of pocket. The COVID-19 response in Malaysia from the onset was centralized and largely coordinated by the Crisis Preparedness and Response Centre (CPRC) under the Ministry of Health Malaysia. As the pandemic persisted and progressed, the number of confirmed cases began to exceed the healthcare capacity of the public sector. The private healthcare sector was then roped in to join efforts in battling the pandemic sustainably as experts began to reconcile with the fact that COVID-19 might be here to stay. One of the main indicators of COVID-19 management capability was reflected in the testing capacity of the country. As of February 7, 2021, a total of 5 215 862 tests had been performed nationwide in Malaysia, with a cumulative positivity rate of 4.65% [31]. This was in line with the criteria published by WHO in May 2020, that a COVID-19 positivity rate of less than 5% is one indicator that the epidemic is under control in a country. As of April 2021, there were a total of 67 COVID-19 laboratories in Malaysia that conduct RT-PCR tests, with 24 being laboratories in the private healthcare centre [32]. Out of those 24 private healthcare laboratories, only 7 were hospital-based, out of which Sunway Medical Centre was one. Together, the healthcare providers from both public and private sector contributed towards the current 70000 per day testing capacity nationwide, as the government worked to further increase the capacity to 150,000 tests per day [33].

Compared to the national average of 4.65% cumulative COVID-19 positivity rate, our study recorded a much lower rate of 1.2% based on the 36603 tests conducted in Sunway Medical Centre from Oct 1, 2020 to Jan 31, 2021. This lower positivity rate correlated with our study finding that majority of those seeking private COVID-19 testing were from low epidemiological risk group and were largely asymptomatic, which decreased their odds of testing positive for COVID-19. Nonetheless, this does not undermine the importance of COVID-19 testing in the private sector, but rather displays its value. A great dilemma faced by governments worldwide in battling the COVID-19 pandemic is in striking a balance between livelihoods and lives–the decision of lifting travel restrictions and reopening economies at the risk of increasing infection rates has stirred public debate. As it takes time for worldwide implementation of COVID-19 immunization program, agencies and governments have resorted to mandating COVID-19 tests to curb spread of disease, with or without symptoms. This screening policy correlated with our study finding which demonstrated that almost 73% of all those seeking COVID-19 testing in our centre performed the test as a mandatory requirement before they were allowed to travel, work or receive medical treatment. Channeling these non-clinically indicated tests to the private sector preserved the testing capacity in the government sector for more medically urgent and high-risk cases, therefore promoting a healthy turnaround time for COVID-19 tests for the nation as a whole. Furthermore, building the testing capacity in the private sector allowed buffering capacity; for the testing burden to be shared during times of sudden increase in positive cases. For example, private laboratories were roped in to help shoulder the burden of clearing backlogged tests during the 3^rd^ and 4^th^ wave of the pandemic, whereby Sunway Medical Centre carried out approximately 2000 tests at subsidized rates between November 2020 to January 2021.

The main limitation of our study is that this was a single-centre study, with a relatively small sample size and lower positivity rate as opposed to the subjects tested nationwide, hence has limited generalizability to Malaysian population. A larger scale study involving multiple institutes from varied geographical locations would be helpful to provide a more holistic view of epidemiological perspectives of COVID-19 in Malaysia.

## Conclusion

In conclusion, we found that loss of smell or taste, fever and running nose were associated with SARS-CoV-2 positivity, consistent with other studies. In addition, we demonstrated that majority of patients seeking COVID-19 testing in private healthcare setting were mainly asymptomatic with low epidemiological risk. The average SARS-CoV-2 positivity rate was 1.2% compared to the national positivity rate of 5.78%. We therefore conclude that most patients who underwent COVID testing in our private institution were of a different demographic group, not provided for by the government COVID-19 testing policy. This finding further illustrates the complimentary role of a private testing facility in providing an effective and comprehensive national screening process as the nation battles against the increasing number of COVID-19 cases.

## Supporting information

S1 Table SARS-CoV-2 positivity rate by reason of screening. (DOCX)
