# Supplementary material for: Clinical and epidemiological characteristics of patients seeking COVID-19 testing in a private centre in Malaysia: Is there a role for private healthcare in battling the outbreak?
Source: PLoS One. 2021 Oct 14;16(10):e0258671. doi: 10.1371/journal.pone.0258671 (PMC8516249; doi:10.1371/journal.pone.0258671)
Supplement: S1 Table — (DOCX) [file pone.0258671.s001.docx]

**Supplementary Table 1. SARS-CoV-2 positivity rate by reason of screening**

| **Reason of Screening** | **Positive (n)** | **Negative (n)** | **Positivity Rate (%)** |
| --- | --- | --- | --- |
| Accident & Emergency | 1 | 68 | 1.45 |
| Admission to hospital | 17 | 6314 | 0.27 |
| Employer requirement | 69 | 7090 | 0.96 |
| On-Site/Workplace screening | 7 | 1204 | 0.58 |
| Pre Travel Requirement | 20 | 11272 | 0.18 |
| Requirement from health authority | 12 | 526 | 2.23 |
| Self-referral - Walk in/Drive Thru/Home Screening | 347 | 9656 | 3.47 |
